# SNPeffect 5.0: large-scale structural phenotyping of protein coding variants extracted from next-generation sequencing data using AlphaFold models

**DOI:** 10.1186/s12859-023-05407-9

**Published:** 2023-07-18

**Authors:** Kobe Janssen, Ramon Duran-Romaña, Guy Bottu, Mainak Guharoy, Alexander Botzki, Frederic Rousseau, Joost Schymkowitz

**Affiliations:** 1 Switch Laboratory, VIB-KU Leuven Center for Brain and Disease Research, Herestraat 49, 3000 Leuven, Belgium; 2 Switch Laboratory, Department of Cellular and Molecular Medicine, KU Leuven, Herestraat 49, 3000 Leuven, Belgium; 3 VIB Bioinformatics Core, VIB, Rijvisschestraat 120, 9052 Ghent, Belgium

**Keywords:** SNPeffect, AlphaFold, Protein stability, Single nucleotide variants, Coding missense variants, Protein aggregation

## Abstract

**Background:**

Next-generation sequencing technologies yield large numbers of genetic alterations, of which a subset are missense variants that alter an amino acid in the protein product. These variants can have a potentially destabilizing effect leading to an increased risk of misfolding and aggregation. Multiple software tools exist to predict the effect of single-nucleotide variants on proteins, however, a pipeline integrating these tools while starting from an NGS data output list of variants is lacking.

**Results:**

The previous version SNPeffect 4.0 (De Baets in Nucleic Acids Res 40(D1):D935–D939, 2011) provided an online database containing pre-calculated variant effects and low-throughput custom variant analysis. Here, we built an automated and parallelized pipeline that analyzes the impact of missense variants on the aggregation propensity and structural stability of proteins starting from the Variant Call Format as input. The pipeline incorporates the AlphaFold Protein Structure Database to achieve high coverage for structural stability analyses using the FoldX force field. The effect on aggregation-propensity is analyzed using the established predictors TANGO and WALTZ. The pipeline focuses solely on the human proteome and can be used to analyze proteome stability/damage in a given sample based on sequencing results.

**Conclusion:**

We provide a bioinformatics pipeline that allows structural phenotyping from sequencing data using established stability and aggregation predictors including FoldX, TANGO, and WALTZ; and structural proteome coverage provided by the AlphaFold database. The pipeline and installation guide are freely available for academic users on https://github.com/vibbits/snpeffect and requires a computer cluster.

**Supplementary Information:**

The online version contains supplementary material available at 10.1186/s12859-023-05407-9.

## Background

Most proteins contain short amino acid stretches that trigger aggregation when becoming solvent-exposed, hence called Aggregation-Prone Regions (APRs) [2, 3]. The presence of a structurally destabilizing mutation can lead to protein unfolding or misfolding and exposure of the APRs. In multi-domain proteins, the exposure of APRs (thus aggregation risk) depends on the convergence of a strong APR with a destabilizing mutation in a specific domain [4]. In addition, mutations can alter the APR strength in a protein, thereby affecting the intrinsic aggregation propensity of a protein.

Next-generation sequencing technologies can identify hundreds to millions of variants depending on the sample. These include coding missense variants, a class of DNA polymorphisms that play an important role as drivers of phenotypic variation and disease. This is particularly true in cancer, where specific driver mutations have been identified that facilitate tumor growth. In addition to driver mutations, cancer cells accumulate many other so-called passenger mutations that are often considered harmless [5]. These can include structurally destabilizing protein mutations with an unknown or unimportant role. The accumulation of many of these variants may impact proteome stability and lead to proteotoxic stress [6]. A recent study showed that cancers with deficient DNA mismatch repair have an increased burden of misfolded protein aggregates, which can be leveraged for immunogenic cell death with immunotherapy [7]. Here, we built an automated bioinformatics pipeline that integrates stability and aggregation predictors for bulk analysis of such variants in a given sample.

## Implementation

### Running SNPeffect 5.0

#### Input file

The pipeline uses the Variant Call Format (VCF) file as input. This standardized text format is used to store variant data, usually acquired by high-throughput sequencing techniques [8].

#### Starting a run

The instructions on how to perform an analysis are described in the README file on the GitHub repository (https://github.com/vibbits/snpeffect). In short, create a working folder that contains the input file (as in.vcf) and then execute the master script (masterscript.pl) to start an analysis. Note that the standard genome used for the pipeline is hg38.

#### Prerequisites

The current pipeline is designed to run on a computer cluster or supercomputer to allow for parallelization since some of the underlying tools require a high computational power (such as FoldX). The software currently only supports the Sun Grid Engine queuing system. However, the master script (masterscript.pl) can be edited to match other cluster configurations.

### Pipeline overview

#### Mapping human variants using SnpEff

**Fig. 1:**
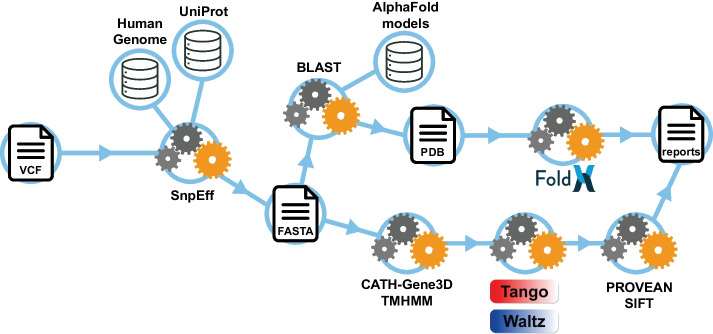
SNPeffect 5.0. Schematic overview of SNPeffect 5.0

A schematic overview of the pipeline is shown in Fig. 1. Starting from a VCF file, the pipeline uses the SnpEff (v5.0) [9] tool for variant annotation and filters for coding missense variants. SnpEff provides all known transcripts for a protein, including different splicing isoforms. Thus, to avoid redundancy, only the transcripts whose sequence matches the UniProt standard reference sequence are kept. FASTA files containing the reference and variant protein sequence are generated. Note that if the user wants to include all the transcripts, this can be adjusted in the master script.

#### Structural stability prediction using FoldX and AlphaFold

The pipeline uses the FoldX (v3.0) [10] force field to predict the variant impact on structural stability. FoldX calculates the free-energy change upon mutation (ΔΔG) using a protein structure as input. The AlphaFold [11, 12] Protein Structure Database is used to provide high structural coverage of the human proteome. Nevertheless, if a BLAST database is provided, the pipeline can also work with experimentally derived PDBs (see documentation). The best matching structure is retrieved by doing a BLAST search of the wild-type sequence against the database. If a protein sequence has more than one perfect match structure, they are all retained. This is the case for AlphaFold structures of very large proteins (> 2700 AA), for which AlphaFold provides overlapping fragments of 1400 AA. AlphaFold produces a per-residue score of its confidence (predicted local distance difference test, pLDDT), which is included in the output file. It is recommended only to consider regions with pLDDT > 70 for reliable structural stability impact predictions. For proteins with more than one structural model, we recommend conducting further analyses using the structure with the highest pLDDT score for the reported variant. In addition, performing energy minimization of the side chains of each PDB structure is highly recommended before modeling with FoldX (see documentation).

#### Domain information (CATH-Gene3D, TMHMM)

Domain information is provided by CATH-Gene3D (v4.3) [13] to allow assessment of the local variant impact on stability and aggregation. It is reported if a variant is present in a specific domain. This is especially useful for multi-domain proteins. The presence of transmembrane regions is predicted using TMHMM (v2.0) [14]. Transmembrane regions are typically rich in hydrophobic residues and are often wrongfully predicted as aggregation-prone regions. The TMHMM annotation allows the filtering of these regions.

#### Aggregation propensity (TANGO and WALTZ)

The next core feature of the pipeline is the prediction of impact on aggregation and amyloid formation tendency using the established predictors TANGO [15] and WALTZ [16], respectively. These tools allow the identification of proteins with high intrinsic aggregation propensity and potential variant impact. The aggregation predictions are reported on the whole protein as well as the specific domain that harbors the amino acid variant.

#### Sequence-based impact predictors (PROVEAN, SIFT)

The sequence-based variant impact predictors PROVEAN (v1.1.5) [17] and SIFT (v6.2) [18] supplement the structure-based stability predictors, which is especially useful for those variants with a low pLDDT score.

#### Output files

The pipeline generates multiple output files including 1) intermediate files that list reasons why particular variants were withheld at a specific step in the analysis and 2) report files that contain the output from the software tools. A detailed description of all output files can be found in the GitHub documentation. In short, the main output files are the SEQANAL and FoldX reports. The SEQANAL report contains all information regarding sequence-based predictors and the FoldX report provides an extensive overview of the variant impact on structural stability. Finally, the ‘finalreport.txt’ gives an overall view of the number of variants that could be mapped and the number of matched structures.

### Pipeline testing

After installation, the user can run the input VCF file from the SHP-77 carcinoma cell line as a test case to verify the correct installation of the software. The input file, intermediate and output files of the test case are provided as a supplement, and the test case is presented in the next section.

## Results and discussion

All versions of SNPeffect have been developed with the specific goal of mapping the effect of missense variants to the protein homeostasis landscape, i.e., the ability of the cell to maintain an appropriate balance of correctly folded proteins. The current version has three significant upgrades that bring it closer to this goal: (1) it allows for high-throughput analysis of variants, (2) it uses AlphaFold structures for high structural coverage of the human proteome, and (3) it provides domain-specific aggregation propensities.

### Case study 1: analysis of all missense variants in dbSNP

To illustrate these three novel aspects of SNPeffect 5.0, we analyzed all missense variants in the dbSNP database that have a defined clinical effect [19]. Since the pipeline requires a VCF file, we manually arranged the variant information to be in VCF format. In total, 277,870 unique missense variants were successfully run with the pipeline starting from the same VCF file (Additional file 1), highlighting its high-throughput capability. In contrast, the previous version of the tool could only analyze one variant at a time, making the study of large datasets impracticable.

**Fig. 2:**
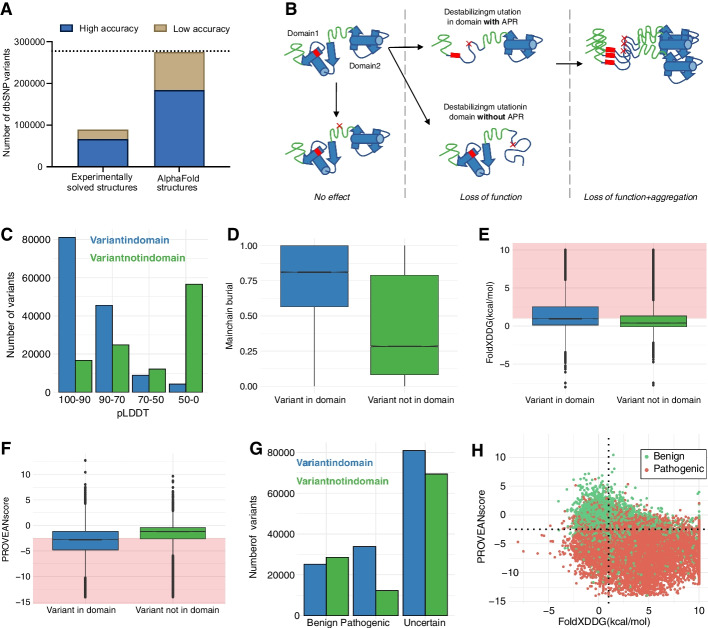
SNPeffect analysis of variants in dbSNP. **A** Number of dbSNP variants that are mapped to a human experimentally solved structure (from PDB) or to an AlphaFold model. High accuracy refers to experimentally solved structures or AlphaFold models with a resolution < 3 Å or a pLDDT score > = 70, respectively. Dotted line indicates the total number of missense variants analyzed. **B** Schematic overview of how mutations can affect a protein based on the context of their structural domains. **C** Number of variants in dbSNP mapped to AlphaFold structures in each pLDDT category. Stability predictions on variants that are in regions with low pLDDT scores (< 70) should be treated with caution. **D** Boxplot showing the mainchain burial of variants in dbSNP. **E** Boxplot of the free-energy change upon mutation (ΔΔG) of dbSNP variants. A ΔΔG > 1 is considered destabilizing for the structure. **F** Boxplot of the pathogenicity impact calculated by PROVEAN upon mutation for dbSNP variants. A PROVEAN score < − 2.5 indicates that the mutation is pathogenic. **G** Number of dbSNP variants in the context of their defined clinical outcome. Variants that are within domains tend to be more pathogenic than variants that are outside domains. **H** Relation of FoldX values and PROVEAN scores. The vertical and horizontal lines represent the FoldX and PROVEAN thresholds for identifying destabilizing or pathogenic variants, respectively

To date, less than half of all human proteins have an experimentally solved structure, and in most cases, it only covers a small fraction of the sequence [20]. In fact, just 33% of the analyzed dbSNP variants can be mapped to a high-resolution experimentally solved structure (Fig. 2A). Thus, using AlphaFold structures, we can obtain nearly complete structural coverage for all variants in dbSNP. The use of FoldX on AlphaFold structures for variant effect prediction was recently shown to provide accurate results [21]. In short, in this study, more than 100,000 mutations from deep mutational experimental measurements were compared with predicted changes in stability for mutations on the AlphaFold structures. The observed correlations are typically as good or better as those obtained with experimentally derived structures when mutants are in regions with a high confidence score (pLDDT score >  = 70). In our example, 67% of all variants are in a region predicted with high confidence, greatly expanding the coverage obtained using experimentally solved structures (Fig. 2A). For regions with a low confidence score, FoldX stability predictions are not reliable [21]. Instead, the output of the sequence-based predictors PROVEAN and SIFT can be used to determine the impact of a variant.

A new feature of SNPeffect 5.0 is the analysis of variants in the context of their structural domains (Fig. 2B). Most proteins contain multiple structural domains that usually fold independently from each other. Therefore, a destabilizing mutation will be, in general, more severe in the domain in which the mutant is located. If the domain contains an APR, the destabilizing mutation will more likely expose it to the solvent and drive protein aggregation [4]. On the other hand, a severe structural destabilization in a domain that does not contain any APRs will generally not result in an aggregation risk to the protein, despite leading to its loss-of-function. Around 51% of analyzed dbSNP variants are mapped to a structural domain by the pipeline. However, variants that are not mapped to domains commonly have low pLDDT scores (Fig. 2C), as they are within linkers. Since these regions are exposed and unstructured [22], variants outside domains are typically not predicted to destabilize or impact the protein’s function, agreeing with their actual clinical phenotype (Fig. 2D–G).

The principles behind structure-based stability predictors, such as FoldX, are very different from sequenced-based predictors, such as PROVEAN or EVE [23]; which might translate into different predicted outcomes for some variants (Fig. 2H). Sequence-based predictors usually rely on a combination of multiple-sequence alignments to estimate the pathogenicity of mutations. However, they do not provide any information on the possible molecular mechanisms of diseases. On the other hand, FoldX uses an empirical force field to determine the change in Gibbs free energy (ΔΔG) of folding upon mutations. Therefore, pathogenic mutations that are mild at the structural level, such as gain-of-function mutations, would not be predicted as destabilizing by FoldX [24]. This distinction is essential, as destabilization can lead to protein unfolding/misfolding and aggregation.

### Case study 2: full exome sequencing of SHP-77 cell line

**Fig. 3:**
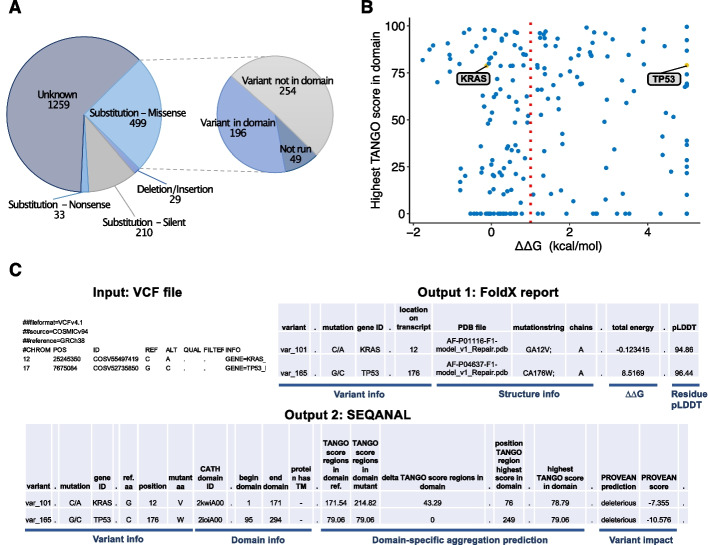
SNPeffect analysis of carcinoma cell line SHP-77.** A** Number of different mutation types in the small cell lung carcinoma cell line SHP-77. The impact of 90% of all missense variants was defined with SNPeffect 5.0. **B** Variant impact plot for the small cell lung carcinoma cell line SHP-77. The APR TANGO score and FoldX scores (maxed at ΔΔG = 5 in the plot) are plotted for all variants in a high-confidence domain. The two variants in known driver proteins, KRAS and p53, are highlighted in the plot. For variants that were matched to more than one PDB structure, only one structure (highest pLDDT) was used to avoid redundancy. **C** Condensed input and output files of cell line SHP-77 focusing on KRAS and p53. The FoldX report contains the calculated impact on structural stability (ΔΔG), information about the used structure, and the residue pLDDT score. The SEQANAL report presents the variant-containing domain, identification of APRs in that domain, and variant-impact prediction by established tools such as PROVEAN. Dotted columns represent extra data that can be found in the complete output files (Additional file 2)

In this section, we emphasize one of the major strengths of the pipeline, which is the capability to analyze all variants present in a sample directly as obtained from next-generation sequencing. This is particularly important in cancer, as the decline in sequencing costs is rapidly moving cancer genomic profiling into routine clinical practice [25]. As an example, we ran the pipeline to all variants identified in the small cell lung carcinoma cell line SHP-77 (downloaded from COSMIC [26]). The output file containing the results can be found in Additional file 2. Out of 499 unique missense variants in this cell line, 450 were successfully run with the pipeline (Fig. 3A). From this, around 45% are within high confidence domains (pLDDT >  = 70) and were used to generate a variant impact plot (Fig. 3B). This plot visualizes the impact on protein stability and convergence with the highest domain TANGO score of all variants in the cell line. Variants that have a destabilizing effect in a protein with a domain containing a strong APR can be found in the upper right quadrant of the plot and can potentially have an additional contribution to proteotoxic stress. SHP-77 cell line contains two variants in known driver proteins with a relatively strong APR (TANGO > 75), KRAS and p53 (Tier 1 Cancer Gene Census [27]) (Fig. 3B, C). A condensed version of the SNPeffect output for the SHP-77 cell line highlighting the specific output of p53 and KRAS is shown in Fig. 3C. Both proteins have a relatively strong APR in the same domain as the variant residue (the highest TANGO score in a domain is 78.9 and 79.1 for KRAS and p53, respectively. In comparison, the oncoprotein (gain-of-function variant) KRAS has a neutral impact on stability (ΔΔG = − 0.12) while tumor suppressor (loss-of-function variant) p53 is severely destabilized (ΔΔG = 8.5) and at risk of misfolding and subsequent aggregation*.* Despite having a neutral impact on stability, the variant affecting KRAS is predicted by PROVEAN to be deleterious (− 7.35) since KRAS G12V is a known oncogenic mutation [28]. Again, this underlines the main difference between using FoldX and a sequence-based variant predictor such as PROVEAN.

The total computational run time for this sample was around 4 h using 25 cores (Intel Xeon Gold 6258R CPU @ 2.70 GHz). In comparison, the computational run time on the same machine with only one core was over 70 h, highlighting the performance increase due to parallelization.

## Conclusions

SNPeffect 5.0 is a novel bioinformatics pipeline for structural phenotyping missense variants directly from sequencing data using stability and aggregation predictors. It offers several major updates to our previous tool versions, including high-throughput analysis, high structural coverage due to the implementation of AlphaFold, and domain-specificity; bringing SNPeffect into the era of high-throughput structural modeling.

## Supplementary Information

**Additional file 1. **Output of the pipeline for all missense variants in dbSNP. 

**Additional file 2. **Output of the pipeline for all missense variants extracted from the SHP-77 cell line.

## Data Availability

The datasets supporting the conclusions of this article are included within the article and its additional files (Additional file 1 & Additional file 2). Project name: SNPeffect 5.0. Project home page: https://github.com/vibbits/snpeffect. Operating systems: Platform independent. Programming language: Perl. Other requirements: The pipeline is built to be used on a computer cluster or supercomputer due to the high computational power needed for some of the underlying tools. The software currently only supports the Sun Grid Engine queuing system. License: The codebase and instruction manual of the pipeline are openly available (MIT license) on the project home page. Any restrictions to use by non-academics: License needed.

## Abbreviations

APR: Aggregation-Prone Region
PDB: Protein Data Bank
VCF: Variant Call Format
pLDDT: Predicted local distance difference test
